# Measuring teamwork and taskwork of community-based “teams” delivering life-saving health interventions in rural Zambia: a qualitative study

**DOI:** 10.1186/1471-2288-13-84

**Published:** 2013-06-27

**Authors:** Kojo Yeboah-Antwi, Gail Snetro-Plewman, Karen Z Waltensperger, Davidson H Hamer, Chilobe Kambikambi, William MacLeod, Stephen Filumba, Bias Sichamba, David Marsh

**Affiliations:** 1 Center for Global Health and Development, Boston University, Boston, MA, USA; 2 Department of International Health, Boston University School of Public Health, Boston, MA, USA; 3 Department of Health and Nutrition, Save the Children, African Region, South Africa; 4 Zambia Center for Applied Health Research and Development, Lusaka, Zambia; 5 Save the Children Zambia, Lusaka, Zambia; 6 Department of Health and Nutrition, Save the Children, Westport, CT, USA

**Keywords:** Teams, Teamwork, Taskwork, Community health workers, Traditional birth attendants, Newborn and child health care, Zambia

## Abstract

**Background:**

The use of teams is a well-known approach in a variety of settings, including health care, in both developed and developing countries. Team performance is comprised of teamwork and task work, and ascertaining whether a team is performing as expected to achieve the desired outcome has rarely been done in health care settings in resource-limited countries. Measuring teamwork requires identifying dimensions of teamwork or processes that comprise the teamwork construct, while taskwork requires identifying specific team functions. Since 2008 a community-based project in rural Zambia has teamed community health workers (CHWs) and traditional birth attendants (TBAs), supported by Neighborhood Health Committees (NHCs), to provide essential newborn and continuous curative care for children 0–59 months. This paper describes the process of developing a measure of teamwork and taskwork for community-based health teams in rural Zambia.

**Methods:**

Six group discussions and pile-sorting sessions were conducted with three NHCs and three groups of CHW-TBA teams. Each session comprised six individuals.

**Results:**

We selected 17 factors identified by participants as relevant for measuring teamwork in this rural setting. Participants endorsed seven functions as important to measure taskwork. To explain team performance, we assigned 20 factors into three sub-groups: personal, community-related and service-related.

**Conclusion:**

Community and culturally relevant processes, functions and factors were used to develop a tool for measuring teamwork and taskwork in this rural community and the tool was quite unique from tools used in developed countries.

## Background

Zambia has high under-five mortality and is not on track to achieve Millennium Development Goal 4, which calls for a two-thirds reduction in under-five mortality from 1990 levels by 2015 [1]. Zambia’s strained health care system with few health facilities and insufficient human resources is inadequate to confront its unacceptably high newborn and under-five mortality [2]. As a consequence of insufficient human resources, many basic health services, especially in rural areas, are provided through several categories of minimally trained community-based providers including community health workers (CHW) and traditional birth attendants (TBA). CHW responsibilities include providing preventive interventions, treatment of common childhood illnesses (fever, diarrhea, and pneumonia), health education and community mobilization and sensitization, as well as supporting outreach services by the rural health staff. TBAs provide maternal and newborn interventions including antenatal care, postnatal care, recognition of and referral for danger signs of pregnant women and newborns. Neighborhood Health Committees (NHCs) selected by the communities support these cadres of health workers as per the Ministry of Health (MOH) community-based delivery strategy [3].

The Center for Global Health and Development (CGHD) of Boston University in partnership with local partners, including the District Health Management Teams (DHMTs) conducted two community-based research projects in Zambia that showed the feasibility and effectiveness of using CHWs and TBAs to provide integrated community case management (CCM) and newborn care [4,5]. Currently TBAs and CHWs may reside in the same community, but work independently of each other, leading to inefficiency and missed opportunities for continuity of care. Experts suggest that health interventions for newborns should be integrated into child health programs [6]. The continuum of care approach is expected to promote care for mothers and children from pregnancy to delivery, the immediate postnatal period and childhood [7].

Save the Children in collaboration with CGHD, the MOH, and the Lufwanyama DHMT is implementing the Lufwanyama Integrated Newborn and Child Health Project in Zambia (LINCHPIN), which teams CHWs and TBAs, supported by NHCs, to provide essential newborn and continuous curative care for children 0–59 months of age in rural Zambia. LINCHPIN is an integrated, community-based newborn care and CCM package delivered through an enhanced district-wide community health program linked to health facilities and NHCs in a manner that is consistent with MOH plans and policies. The rationale for the integration and the teamwork is to close the gap in the continuum of care and increase the likelihood that the effect of the team will exceed the effects of the individuals working alone.

Teams occur in many settings, including health care, in both developed and developing countries. There is a general agreement that a team consists of two or more individuals who have specialized knowledge, have specific roles, make decisions, perform interdependent tasks, are adaptable, and share a common goal [8-10]. Benefits of a team may include distributing workload among team members, reinforcing individual capabilities, creating the feeling of participation and involvement, better decision-making and generating a diversity of ideas for a common purpose [11]. Two general categories of behaviors are often used to distinguish a team: teamwork and taskwork. Teamwork consists of behaviors that are related to team member interactions and are necessary to establish coordination among individual team members in order to achieve team goals whereas taskwork consists of behaviors that are performed by individual team members and are critical to the execution of individual team member functions [12,13].

Assessments of the impact of teamwork have occurred in medical settings such as operating rooms [14] and emergency departments [15]; furthermore, teamwork has been linked to patient safety [16] in well-resourced settings. Measuring teamwork to ascertain whether the team is performing as expected to achieve the desired output is rare in health care settings in developing countries. Our review of the literature revealed one report in which the MOH and *Médecins sans Frontières* formed community health teams comprised of community health agents, community health volunteers and TBAs in Mozambique’s Angónia District to improve coverage of basic health services including tuberculosis and HIV care [17]. Team members received joint five-day initial training and were provided the necessary drugs, supplies and job aides. Although the report lacked measures of teamwork or evidence of effect at the beneficiary level, the authors asserted that the teams had advantages over a “vertical CHW” approach in the areas of mutual accountability, joint problem-solving, improved delivery of preventive and curative health services, and consistent health education messages. They concluded that the team approach improved accountability, acceptability, and access to care.

In cases where teamwork has been measured, dimensions of teamwork or processes that comprise the teamwork construct such as: goal comprehension, communication, conflict management, decision-making/planning, leadership, mutual performances monitoring, mutual trust, team cohesion and team motivation have been used, [10,16,18-20]. This paper describes the process of developing a measure of teamwork and also taskwork for community-based health teams in rural Zambia.

## Methods

### Study location

The study was conducted in Lufwanyama District in the Copperbelt Province of Zambia. Lufwanyama is a large, rural, undeveloped district with a population of 85,033 [21]. Despite its location in the comparatively urban, industrialized Copperbelt, the district lacks physical infrastructure, and most roads are frequently impassible during the rainy season. It has 11 health centers and four health posts, but no district hospital - indeed the district health office is currently outside the district pending completion of a new district seat. Many basic health services including treatment of minor illnesses, health education, antenatal care, family planning services, follow up of patients with chronic illnesses and referrals are provided through several categories of minimally trained community workers –TBAs, CHWs, male motivators, safe motherhood agents, family planning agents, disease surveillance agents, malaria agents, tuberculosis agents, HIV/AIDS agents, as well as untrained TBAs.

### Study design

This formative research employed a qualitative methodology using a combination of group discussion and pile-sorting to explore and identify processes and domains for measuring teamwork and functions for measuring taskwork. The pile sorting technique engages participants in sorting cards with words into piles that represent how they think about and categorize elements on interest [22]. Six sessions were conducted, three with NHC members and three with CHW-TBA pairs. Each NHC session was made up of the chairperson, the secretary and four other members including at least two women. The CHW –TBA sessions were made up of three CHWs and three TBAs. We purposively selected three NHCs considered as “highly effective” by the DHMT (held regular meetings and had strong, dynamic chairpersons). The CHWs and TBAs came from the selected NHC areas. A total of 36 individuals were involved. This number may be small but sample sizes of 30–40 have been shown to have adequate reliability and found acceptable for validity in card sorting tasks [23,24].

### Group discussions and pile sorting

Each session conducted in the form of a focus group discussion (FGD) had a facilitator and a recorder and was held at a quiet place in the community lasting about 1.5 to 2 hours. The session was audio-recorded, and the recorder also took written notes of the discussions. All sessions were facilitated in the local language, Bemba.

Each session had three parts.

The first part was a group discussion. We used a discussion guide with open-ended questions and a time-line activity to identify local concepts, perceptions and experiences of teamwork processes. The guide was pretested to ensure that the questions were clear and understandable to the people involved since the guide was translated into the local language. The timeline activity initiated dialogue on teamwork. Participants were asked to give examples of a recent situation where they worked with someone else to help mothers and children stay healthy. The events were plotted on a timeline on the ground using sticks, stones, and leaves. Probe questions included: *How or why did you decide to invite someone to help you? What was the first thing this person did to help? What was the next thing they did? Looking back on this timeline, what was the most helpful thing this person did? Why do you think you worked well as a team? What would have made this team work better? What made your team work well? Now, share a time when the team’s work did not go as expected? What made it not go well? What could have improved the team’s work?* The same guide was used in all the six sessions and the questions were asked in the same order.

During the discussions, participants were asked to identify processes that helped or hindered teamwork. The processes that participants indicated as important for teamwork were written on cards by the facilitator. We wrote cards ahead of time of processes (from the literature, our experience and pre-formative discussion with the community) that we consider as important for teamwork. The purpose was for the facilitator to ask the participants if these processes were not mentioned in the discussion to indicate whether they were important for teamwork.

The second part was the pile sorting, during which the processes written on cards were then sorted. Participants were given the cards and asked to work as a team to sort the cards into three groups: “very important”, “important” and “least important”. After the sorting, the facilitator took each of the cards in the “very important” group and asked the participants to explain why they considered it as “very important”. The reasons given were recorded by the note taker.

During the third part, a list of seven functions prepared prior to the sessions by the investigators through consultation with health workers, community based workers and NHCs was introduced. The purpose was to ascertain whether the participants agree that TBAs and CWHs need to jointly perform these pre-determined functions so that they could be incorporated into the tool to measure taskwork. We asked participants to indicate and explain which of the functions they considered important for the CHW and TBA to perform jointly in order to assist them in providing life-saving integrated newborn care and CCM interventions.

### Data analysis

We used a weighting system to select factors for measuring teamwork from those identified and sorted by the participants. Five points were given for “very important”, three for “important” and one for “least important”. A factor was selected if it scored 22 or more points out of a possible 30 points. We chose a score of at least 22 to ensure that a factor is selected if at least two FGDs indicated it as “very important” and the reaming four FGDS indicated it as “important”. We further categorized the selected factors into dimensions of teamwork, or processes that comprise the teamwork construct. There were some factors which were identified and sorted by the participants but which we thought that they do not necessarily measure teamwork but rather may influence the way the team performs. These were termed as determinants and may explain why teams engage in effective teamwork. We categorized these factors (determinants) into three groups: personal, community-related and service-related.

### Ethical issues

Ethical approval was obtained from the Boston University Institutional Review Board (BU-IRB) and Zambia’s ethical review committee (ERES CONVERGE). Informed consent was obtained from all study participants. A consent form developed in accordance with guidelines of the BU-IRB and the local ethical review committee was translated into Bemba, the local language.

## Results

### Participant characteristics

The NHC participants included 12 males and 6 females. Male participants were older than female participants (average age 46.9 [range 34–59] vs. 35.5 years [range 28–53]) and had attained higher education levels than their female counterparts (Grade 10 and above: 70% vs. 33%). All NHC participants were farmers except for two female members who were business women. CHW-TBA participants comprised 7 males and 11 females. Two CHWs and all the TBAs were females. TBAs were older than the CHWs (average age 52.6 [range 46–58] vs. 46.5 years [range 35–65]). CHWs were more educated than the TBAs. All CHWs had attained grade 9 or above while most TBAs had only reached grade 7 or below. Two TBAs had no schooling. All CHWs and TBAs were farmers.

### Processes and factors for teamwork

Seventeen factors identified by the participants that scored 22 or more were selected to measure teamwork. We categorized these factors into dimensions of teamwork or processes that comprise the teamwork construct (Table 1). All the six FGDs identified three of the 17 factors as “very important,” and five FGDs identified six as “very important”. One factor “motivating each other” was considered “very important” by only two of the six groups, one NHC and the other CHW-TBA. Two groups (one NHC and the other CHW-TBA) considered all the seventeen factors as “very important” for measuring teamwork. Factors which scored below 22 and therefore not selected included “leadership”; “similar vision”, “mutual support” and “coordination among members”. All six FGDs indicated that leadership was not important in a two person team. Reasons participants sorted some of the factors into the “very important” group are shown in Table 2.

**Table 1:** Processes and factors of teamwork

**Process**	**Factors**
1. Mutual performance monitoring	1) Consulting each other
	2) Seeking help from each other
	3) Checking each other’s work and giving feedback
2. Mutual trust	4) Confidentiality
	5) Respect
	6) Trust
3. Decision making/planning	7) Making decisions together
	8) Making a plan together
	9) Dividing tasks so not to duplicate effort
4. Team cohesion	10) Interest and commitment
	11) Members available and accessible
5. Team motivation	12) Motivating each other
	13) Encouraging each other
6. Goals and objectives	14) Having a common goal
7. Communication	15) Good communication
	16) Sharing information
8. Conflict resolution/management	17) Ability to manage conflict

**Table 2:** Importance and illustrative quotations of teamwork factors

**Factors**	**# Groups indicating factor as “very important”**	**Illustrative Quotation**
Confidentiality	6	• *Many NHCs have stopped functioning because there was lack of confidentiality among members.*
		• *Many mothers refused to go to CHWs because of lack of confidentiality.*
		• *If there is no confidentiality among us as team members, the community will be scared to access the needed services from us.*
		• *Lack of confidentiality in a team can lead to dismantling of the team.*
Having a common goal	6	• *A common goal gives direction to a team.*
		• *A team without a common goal has no direction.*
Making a plan together	6	•*Making a plan together is the ingredient for achieving the goal of a team*
Good communication	5	• *Anytime we do not communicate among ourselves, we feel our team is collapsing.*
Seeking help from each other	4	• *If we cannot help each other when the need arises, how can we work together? It’s like going in different directions*.
Members available and accessible	4	• *How can you work as a team if members are not available when needed?*
Checking each other’s work and giving feedback	4	• *It is important to learn from each other what happened, our mistakes and successes.*
		• *If we are not giving feedback, how can we learn from the past?*
		• *Not learning from the past will affect the performance of the team.*
Dividing tasks so not to duplicate effort	4	• *Duplicating efforts can cause conflict in the team.*

### Jointly performed functions for taskwork

Participants indicated that all of the seven pre-determined functions presented to them were essential for the CHWs and TBAs to perform jointly if they were to provide life-saving integrated newborn care and CCM interventions effectively. The functions were:

1. Joint monthly meetings with NHCs to discuss work and performance.

2. Joint behavior change communications sessions targeting women on newborn and child care.

3. Joint problem solving with regard to newborn or child care.

4. Joint participation in outreach services including child welfare clinics and immunization conducted by the supervising rural health center staff.

5. Collaboration to refer a pregnant woman or a mother with a sick child to the rural health center or hospital if necessary.

6. Intra-team referral (referral between team members, for example, CHW referring a pregnant woman to the TBA or TBA referring a mother with a sick child 0–59 months to the CHW).

7. Joint postnatal care visits to a mother with a newborn aged about 6–8 weeks where the TBA “hands over” the child to the CHW.

We used these functions to measure taskwork.

### Determinants of teamwork

We selected 20 factors identified by the participants as determinants of teamwork. These factors may explain why teams engage in effective teamwork. We categorized these factors into three sub-groups: personal, community-related and service-related. Most of the factors belonged to the personal and service-related sub-groups (Table 3).

**Table 3:** Factors for measuring the determinants of teamwork

**Personal**	**Community–related**	**Service-related**
• Age	• Presence of and links to NHCs	• Training
• Gender	• Distance between CHW and TBA families	• Experience
• Education	• Distances among , CHW and rural health center	• Supervision and support by relevant community and health system structures
• Socio-economic status		• Payment or in-kind compensation
• Language		• Motivation
• Tribal affiliation		• Availability of means of transport (e.g. bicycle)
• Religion		• Possession of a cell phone
•Employment		• Availability of various supplies and drugs that the CHW and TBA might need to provide the defined services
• Membership in an association		

## Discussion

This formative research employing group discussion and pile sorting enabled community-generated processes, functions and factors to be elicited to measure teamwork and taskwork, and determinants of teamwork in this setting. We used this methodology because of its ability to promote consensus among group members [22]. Pile sorting has been used in public health settings to capture local definitions of disease [25,26], to study relationships between symptoms and disease severity [27]; and to investigate the acceptability of interventions [28,29]. In our case the pile sorting was constrained, as participants organized the cards according to categories provided to them [30]. Relatively few studies have used pile sorting in focus groups similar to ours [31,32].

The 17 factors identified for measuring teamwork were categorized under eight of the processes that comprise teamwork construct: 1) mutual performance monitoring, 2) mutual trust, 3) decision making/planning, 4) team cohesion, 5) team motivation, 6) goals and objectives, 7) communication and 8) conflict resolution/management. Three of our processes were included in the Team Development Measure constructed by Mahoney and Turkovich to measure the level of development of a team in health care setting in the developed world [18]. Communication was also part of the TeamSTEPPS Teamwork Attitudes Questionnaire, a measure designed to assess attitudes towards the core components of teamwork in healthcare [10]. Factors that affect a team’s processes identified by a WHO Working group on patient safety [16] were similar to what we found.

Most of the seventeen factors we identified for measuring teamwork belong to teamwork attitudes and behaviors and this underscores their importance in team performance in this rural setting. Leadership, commonly an important construct for measuring teamwork, was considered unimportant in this setting. Indeed, participants indicated that the team would likely fail if one member imposes him/herself as a leader of the team, perhaps because of team composition and small size and/or the relatively egalitarian rural culture. The seven functions identified for measuring taskwork emphasize the importance of strong relationship between the community-based workers and the community leadership in charge of health on one hand, and the community-based workers and the beneficiaries of their services on the other.

The 20 factors identified as determinants of teamwork will assess the relationship between the level of team performance and personal, service-related and community-related factors. Community and social systems are often integrated and linked; therefore assessing the relationship between the level of teamwork and these determinants, especially the community related determinants such as the supportive role of the NHC to the CHW/TBA team is important. The personal factors include age and gender which research in developed world has not typically found to have any relationship with teamwork. We however think since we are dealing with a rural community where age and gender are very sensitive issues and our teams are composed of two persons, these factors may be important.

The developed tool (Additional file 1) has three parts. Part A is administered to both the CHW and TBA jointly and measures taskwork. It assesses whether the team jointly performs and documents the seven functions in the previous three months. The team scores “0” if a function is not performed, “1” if performed but there is no documentary evidence and “2” if there is documentary evidence. Part B is administered separately to the CHW and the TBA and measures teamwork through 27 characteristics/indicators derived from the17 factors selected for teamwork. This elicits the team’s opinion whether the characteristic is present in their team over the previous six months. Each characteristic has three responses “No” or “never; ii) “sometimes” and iii) “Yes” or “all the time” and the scores 1, 2 and 3 respectively. The score for the team is the average score of the two members. Part C collects information on the determinants of teamwork and is administered separately to each individual team member to explain why teams engage in effective teamwork.

The tool is intended to be used by the supervisors (the rural health center staff and the DHMT) of the community-based workers to assess the level of teamwork and taskwork and their relationship to the utilization of the services being provided by the teams. The processes of teamwork and taskwork functions represent unique skills, and together form integral part of an effective community based team. These processes and functions can serve as competencies to be strengthened during refresher trainings to improve team performance.

This tool is unique that it measures community based healthcare volunteers’ views of teamwork and taskwork. Most of the existing tools are not aligned with what the literature advocates as the core components of teamwork. For example, the Safety Climate Survey tool measures perceptions of organizational commitment to patient safety such as commitment to safety, leadership, interpersonal interactions, attitudes towards stress and knowledge of how to report adverse events [33]. The Safety Attitudes Questionnaire also measures attitudes about teamwork climate, safety climate, perceptions of management, job satisfaction, working conditions and stress [34]. Another tool, the Team Climate Assessment Measurement Questionnaire was developed to enable teams in health and social care to review aspects of their team that are believed to affect patient safety and error management [35].

A limitation of this study was the purposive selection of well-functioning NHCs. We needed to be able to draw on “functional” NHC prior experience working with community members to solve health problems and identify existing “best practices”. This was essential because there would be no point in studying a disorganized, dysfunctional setting where teamwork was unlikely to have been present. We also acknowledge the complexity of measuring some of the determinants such as socio-economic status, motivation and links with NHCs. Another limitation of the study is the small number of participants.

## Conclusion

To our knowledge, this is the first tool developed to assess teamwork and taskwork in a community-based health care setting in a developing country, and the first tool to assess a two-person team. We used a qualitative participatory methodology involving the population (community health workers and committees) the tool is targeted for in the process of developing the tool. We believe that this approach may contribute to making the tool acceptable to the target population. The method was simple and proved highly valuable for identifying community and culturally relevant processes for measuring teamwork and functions for measuring taskwork. The simplicity of this method and its value in identifying community- and culturally-relevant processes and functions are strengths of this approach. We believe our tool can be adapted to measure teamwork and taskwork in other health settings and in situations where there are more than two members of a team.

## Abbreviations

BU-IRB: Boston University Institutional Review Board; CCM: Community Case Management; CGHD: Center for Global Health and Development; CHW: Community Health Worker; DHMT: District Health Management Team; LINCHPIN: Lufwanyama Integrated Newborn and Child Health Project; MOH: Ministry of Health; NHC: Neighborhood Health Committee; TBA: Traditional Birth Attendant

## Competing interests

All other authors declare that they have no competing interests.

## Authors’ contributions

KYA, DM, DHH, KYA, KZW, and WM contributed to the conception and design of the study. KYA, DHH, GSP, CK, SF, and BS all participated in study implementation and data collection. KYA, DHH and WM performed data analyses and with DM, GSP and KZW assisted with interpretation of the data. KYA, DHH and DM drafted the manuscript. All authors contributed to revisions of the manuscript and read and approved the final manuscript.

## Pre-publication history

The pre-publication history for this paper can be accessed here:

http://www.biomedcentral.com/1471-2288/13/84/prepub

## Supplementary Material

Additional file 1 Team Measurement Tool. Click here for file
