# Supplementary material for: Measuring teamwork and taskwork of community-based “teams” delivering life-saving health interventions in rural Zambia: a qualitative study
Source: BMC Med Res Methodol. 2013 Jun 27;13:84. doi: 10.1186/1471-2288-13-84 (PMC3698032; doi:10.1186/1471-2288-13-84)
Supplement: Additional file 1 — Team Measurement Tool. [file 1471-2288-13-84-S1.docx]

**PART A: TASKWORK (To be administered to both members jointly)**

**Introduction**

The following statements describe functions and responsibilities you are supposed to perform as a team. Please answer all questions as openly and honestly as possible.

A1. Has this team jointly attended an NHC meeting in the last 3 months?

| 1. No |
| --- |
| 2. Yes but no documentation |
| 3. Yes and documented |

A2. Has this team jointly conducted BCC (health education) session on newborn or child care in this community in the last 3 months?

| 1. No |
| --- |
| 2. Yes but no documentation |
| 3. Yes and documented |

A3. Has this team jointly worked together to solve any problem related to newborn or child care in this community in the last 3 months?

| 1. No |
| --- |
| 2. Yes but no documentation |
| 3. Yes and documented |

A4. Has this team jointly participated in outreach services in this community in the last 3 months?

| 1. No |
| --- |
| 2. Yes but no documentation |
| 3. Yes and documented |

A5. Has this team jointly worked together to refer a pregnant woman or a mother with a sick child 0 – 59 months to the health center/post in the last 3 months?

| 1. No |
| --- |
| 2. Yes but no documentation |
| 3. Yes and documented |

A6. Has there been any referral between team members (for example, CHW referring a pregnant woman to the TBA or TBA referring a mother with a child 0-59 months to the CHW) in the last 3 months?

| 1. No |
| --- |
| 2. Yes but no documentation |
| 3. Yes and documented |

A7. Has the team jointly conducted PNC visit to a mother with a newborn aged about 6-8 weeks where the TBA handed over the newborn to the CHW in the last 3 months?

| 1. No |
| --- |
| 2. Yes but no documentation |
| 3. Yes and documented |

**PART B: TeamWORK (To be administered to only the CHW)**

**Introduction**

The following statements describe certain features and characteristics that may be present in your team. Please indicate what most represents the current situation for your team.

Please answer all questions as openly and honestly as possible.

CB1. Do you make plans together towards achieving the goal of this team which is improving the health of children?

| 1. No |
| --- |
| 2. Sometimes |
| 3. All the time |

CB2. Do you and your team member (TBA) clearly understand your common goal?

| 1. No |
| --- |
| 2. Not sure |
| 3. Yes |

CB3. Do you and your team member (TBA) clearly understand your roles and responsibilities in this team work?

| 1. No |
| --- |
| 2. Not sure |
| 3. Yes |

CB4. Do you make decisions together about the work of your team?

| 1. No |
| --- |
| 2. Sometimes |
| 3. All the time |

CB5. Do you divide your tasks so as not to duplicate efforts?

| 1. No |
| --- |
| 2. Sometimes |
| 3. All the time |

CB6. Do you check each other’s work to ensure that you are each doing what you are expected to achieve your goals?

| 1. No |
| --- |
| 2. Sometimes |
| 3. All the time |

CB7. Are you and your team member (TBA) working together to achieve the goal of improving the health of children?

| 1. No |
| --- |
| 2. Not sure |
| 3. Yes |

CB8. Do you think there is mutual respect between you and your team member (TBA)?

| 1. No |
| --- |
| 2. Not sure |
| 3. Yes |

CB9. Do you feel there is mutual trust rather than suspicion or anxiety in your team?

| 1. No |
| --- |
| 2. Not sure |
| 3. All the time |

CB10. Do you feel that issues you deal with as a team are strictly confidential?

| 1. No |
| --- |
| 2. Not sure |
| 3. Yes |

CB11. Do you work through disagreements or conflicts with your team member (TBA) to manage them when they arise?

| 1. No |
| --- |
| 2. Sometimes |
| 3. All the time |
| 4. No disagreement/conflict |

CB12. Do you enjoy working together with your team member (TBA)?

| 1. No |
| --- |
| 2. Sometimes |
| 3. Yes |

CB13. How committed are you to the success of this team?

| 1. No commitment |
| --- |
| 2. Some commitment |
| 3. Very committed |

CB14. How often do you communicate with your team member (TBA)?

| 1. Never |
| --- |
| 2. Sometimes |
| 3. Very often |

CB15. How available and accessible are you to support your team member (TBA) when there is the need?

| 1. Never |
| --- |
| 2. Sometimes |
| 3. All the time |

CB16. How often do you consult your team member (TBA) when there is the need?

| 1. Never |
| --- |
| 2. Sometimes |
| 3. All the time |

CB17. Do you seek help from your team member (TBA) if there is the need?

| 1. No |
| --- |
| 2. Sometimes |
| 3. All the time |

CB18. Do you openly share with your team member (TBA) information important for the success of the team?

| 1. No |
| --- |
| 2. Sometimes |
| 3. All the time |

CB19. Do you feel that the teamwork is worthwhile?

| 1. No |
| --- |
| 2. Not sure |
| 3. Yes |

CB20. Do you have a strong sense of being a member of this team?

| 1. No |
| --- |
| 2. Not sure |
| 3. Yes |

CB21. Do you motivate your team member (TBA) to work as a team?

| 1. No |
| --- |
| 2. Sometimes |
| 3. All the time |

CB22. Do you encourage your team member (TBA) to perform agreed upon roles and responsibilities in the team?

| 1. No |
| --- |
| 2. Sometimes |
| 3. All the time |

CB23. Do you feel free to share your ideas with your team member (TBA) about how the work is going and how to improve upon it?

| 1. No |
| --- |
| 2. Sometimes |
| 3. Yes |

CB24. Do you feel free to express your feelings with your team member (TBA) about how the work is going and how to improve upon it?

| 1. No |
| --- |
| 2. Sometimes |
| 3. Yes |

CB25. Do you feel that your team member (TBA) takes over what you consider to be your role?

| 1. Yes |
| --- |
| 2. Sometimes |
| 3. No |

CB26. Do you feel that your team member (TBA) interferes with your work?

| 1. Yes |
| --- |
| 2. Sometimes |
| 3. No |

CB27. Do you feel that your team member (TBA) is trying to control the team?

| 1. Yes |
| --- |
| 2. Sometimes |
| 3. No |

**PART B: TeamWORK (To be administered to only the TBA)**

**Introduction**

The following statements describe certain features and characteristics that may be present in your team. Please indicate what most represents the current situation for your team.

Please answer all questions as openly and honestly as possible.

TB1. Do you make plans together towards achieving the goal of this team which is improving the health of children?

| 1. No |
| --- |
| 2. Sometimes |
| 3. All the time |

TB2. Do you and your team member (CHW) clearly understand your common goal?

| 1. No |
| --- |
| 2. Not sure |
| 3. Yes |

TB3. Do you and your team member (CHW) clearly understand your roles and responsibilities in this team work?

| 1. No |
| --- |
| 2. Not sure |
| 3. Yes |

TB4. Do you make decisions together about the work of your team (CHW)?

| 1. No |
| --- |
| 2. Sometimes |
| 3. All the time |

TB5. Do you divide your tasks so as not to duplicate efforts?

| 1. No |
| --- |
| 2. Sometimes |
| 3. All the time |

TB6. Do you check each other’s work to ensure that you are each doing what you are expected to achieve your goals?

| 1. No |
| --- |
| 2. Sometimes |
| 3. All the time |

TB7. Are you and your team member (CHW) working together to achieve the goal of improving the health of children?

| 1. No |
| --- |
| 2. Not sure |
| 3. Yes |

TB8. Do you think there is mutual respect between you and your team member (CHW)?

| 1. No |
| --- |
| 2. Not sure |
| 3. Yes |

TB9. Do you feel there is mutual trust rather than suspicion or anxiety in your team?

| 1. No |
| --- |
| 2. Not sure |
| 3. All the time |

TB10. Do you feel that issues you deal with as a team are strictly confidential?

| 1. No |
| --- |
| 2. Not sure |
| 3. Yes |

TB11. Do you work through disagreements or conflicts with your team member (CHW) to manage them when they arise?

| 1. No |
| --- |
| 2. Sometimes |
| 3. All the time |
| 4. No disagreement/conflict |

TB12. Do you enjoy working together with your team member (CHW)?

| 1. No |
| --- |
| 2. Sometimes |
| 3. Yes |

TB13. How committed are you to the success of this team?

| 1. No commitment |
| --- |
| 2. Some commitment |
| 3. Very committed |

TB14. How often do you communicate with your team member (CHW)?

| 1. Never |
| --- |
| 2. Sometimes |
| 3. Very often |

TB15. How available and accessible are you to support your team member (CHW) when there is the need?

| 1. Never |
| --- |
| 2. Sometimes |
| 3. All the time |

TB16. How often do you consult your team member (CHW) when there is the need?

| 1. Never |
| --- |
| 2. Sometimes |
| 3. All the time |

TB17. Do you seek help from your team member (CHW) if there is the need?

| 1. No |
| --- |
| 2. Sometimes |
| 3. All the time |

TB18. Do you openly share with your team member (CHW) information important for the success of the team?

| 1. No |
| --- |
| 2. Sometimes |
| 3. All the time |

TB19. Do you feel that the teamwork is worthwhile?

| 1. No |
| --- |
| 2. Not sure |
| 3. Yes |

TB20. Do you have a strong sense of being a member of this team?

| 1. No |
| --- |
| 2. Not sure |
| 3. Yes |

TB21. Do you motivate your team member (CHW) to work as a team?

| 1. No |
| --- |
| 2. Sometimes |
| 3. All the time |

TB22. Do you encourage your team member (CHW) to perform agreed upon roles and responsibilities in the team?

| 1. No |
| --- |
| 2. Sometimes |
| 3. All the time |

TB23. Do you feel free to share your ideas with your team member (CHW) about how the work is going and how to improve upon it?

| 1. No |
| --- |
| 2. Sometimes |
| 3. Yes |

TB24. Do you feel free to express your feelings with your team member (CHW) about how the work is going and how to improve upon it?

| 1. No |
| --- |
| 2. Sometimes |
| 3. Yes |

TB25. Do you feel that your team member (CHW) takes over what you consider to be your role?

| 1. Yes |
| --- |
| 2. Sometimes |
| 3. No |

TB26. Do you feel that your team member (CHW) interferes with your work?

| 1. Yes |
| --- |
| 2. Sometimes |
| 3. No |

TB27. Do you feel that your team member (CHW) is trying to control the team?

| 1. Yes |
| --- |
| 2. Sometimes |
| 3. No |

**PART C: determinants OF TEAMWORK (To be administered to only the CHW)**

CH1a. Have you received any supervision in your work from the rural health center/DHMT in the last 3 months?

| 1. No | 2. Yes |
| --- | --- |

CH1b. Where did it take place?

| 1. At the community health post |
| --- |
| 2. At the health facility |
| 3. At your home |
| 4. Other______________________________ |
| 8. NA |

CH1c. Was the supervision for both you and your team?

| 1. No | 2. Yes | 8. NA |
| --- | --- | --- |

CH1d. Were you supervised as a team together at the same time?

| 1. No | 2. Yes | 8. NA |
| --- | --- | --- |

CH2. The last time you were personally supervised, did your supervisor do any of the following?

| CH2.1 | Deliver supplies | 1. Yes | 2. No | 8. NA |
| --- | --- | --- | --- | --- |
| CH2.2 | Check/review your records/registers | 1. Yes | 2. No | 8. NA |
| CH2.3 | Observe you working | 1. Yes | 2. No | 8. NA |
| CH2.4 | Provide any feedback/comments that you are doing your work well | 1. Yes | 2. No | 8. NA |
| CH2.5 | Provide any feedback/comments that you need improvement in one or more areas | 1. Yes | 2. No | 8. NA |
| CH2.6 | Provide updates on technical issues related to your work | 1. Yes | 2. No | 8. NA |
| CH2.7 | Discuss problems you have encountered | 1. Yes | 2. No | 8. NA |

CH3. Have you received any payment, cash or/and in kind for the work you do in the last six months?

| 1. No |
| --- |
| 2. Cash only |
| 3. In kind only |
| 4.Both cash and in kind |

CH4. How satisfied are you with your work as a CHW?

| 1. Not satisfied |
| --- |
| 2. Somewhat satisfied |
| 3. Satisfied |
| 4. Highly satisfied |

CH5. How motivated are you in performing your work as CHW?

| 1. Not motivated |
| --- |
| 2. Somewhat motivated |
| 3. Motivated |
| 4. Highly motivated |

| CH6. Do you own a bicycle? | 1. No | 2. Yes |
| --- | --- | --- |

| CH7. Do you own a cell phone? | 1. No | 2. Yes |
| --- | --- | --- |

CH8. Have you received any refresher training on newborn or child care in the last six months?

| 1. No | 2. Yes |
| --- | --- |

CH9. Have you had any discussion with community leaders or other community groups (not NHCs) about your work in the last six months?

| 1. No | 2. Yes |
| --- | --- |

CH10. CHECK THE AVAILABILITY OF DRUGS/SUPPLIES

| CH10.1 | ACT (Coartem/Lumet) | 1. Yes | 2. Not available today | 3. Never available |
| --- | --- | --- | --- | --- |
| CH10.2 | Amoxicillin for pneumonia | 1. Yes | 2. Not available today | 3. Never available |
| CH10.3 | ORS packets | 1. Yes | 2. Not available today | 3. Never available |
| CH10.4 | Zinc | 1. Yes | 2. Not available today | 3. Never available |

**PART D: determinants OF TEAMWORK (To be administered to only the TBA)**

D1a. Have you received any supervision in your work from the rural health center/DHMT in the last six months?

| 1. No | 2. Yes |
| --- | --- |

D1b. Where did it take place?

| 1. At the community health post |
| --- |
| 2. At the health facility |
| 3. At your home |
| 4. Other______________________________ |
| 8. NA |

D1c. Was the supervision for both you and your team member?

| 1. No | 2. Yes | 8. NA |
| --- | --- | --- |

D1d. Were you supervised as a team together at the same time?

| 1. No | 2. Yes | 8. NA |
| --- | --- | --- |

D2. The last time you were personally supervised, did your supervisor do any of the following?

| D2.1 | Deliver supplies | 1. Yes | 2. No | 8. NA |
| --- | --- | --- | --- | --- |
| D2.2 | Check/review your records/registers | 1. Yes | 2. No | 8. NA |
| D2.3 | Observe you working | 1. Yes | 2. No | 8. NA |
| D2.4 | Provide any feedback/comments that you are doing your work well | 1. Yes | 2. No | 8. NA |
| D2.5 | Provide any feedback/comments that you need improvement in one or more areas | 1. Yes | 2. No | 8. NA |
| D2.6 | Provide updates on technical issues related to your work | 1. Yes | 2. No | 8. NA |
| D2.7 | Discuss problems you have encountered | 1. Yes | 2. No | 8. NA |

D3. Have you received any payment, cash or/and in kind for the work you do in the last six months?

| 1. No |
| --- |
| 2. Cash only |
| 3. In kind only |
| 4.Both cash and in kind |

D4. How satisfied are you with your work as a TBA?

| 1. Not satisfied |
| --- |
| 2. Somewhat satisfied |
| 3. Satisfied |
| 4. Highly satisfied |

D5. How motivated are you in performing your work as TBA?

| 1. Not motivated |
| --- |
| 2. Somewhat motivated |
| 3. Motivated |
| 4. Highly motivated |

| D6. Do you own a bicycle? | 1. No | 2. Yes |
| --- | --- | --- |

| D7. Do you own a cell phone? | 1. No | 2. Yes |
| --- | --- | --- |

D8. Have you received any refresher training on newborn or child care in the last six months?

| 1. No | 2. Yes |
| --- | --- |

D9. Have you had any discussion with community leaders or other community groups (not NHCs) about your work in the past six months?

| 1. No | 2. Yes |
| --- | --- |

D10. CHECK THE AVAILABILITY OF SUPPLIES

| D10.1 | TBA Kits/some materials for delivery or newborn care | 1. Yes | 2. Not available today | 3. Never available |
| --- | --- | --- | --- | --- |
